# A Novel Do-It-Yourself Approach to Simulating Minimally Invasive Laparoscopic Surgery

**DOI:** 10.7759/cureus.38510

**Published:** 2023-05-03

**Authors:** Jeremy Wright, Andrew M Joseph, Khloud Yassin, Carlos Fagundo, Marshall Aske, Lohitha Guntupalli, Rudresh Patel, Heather McKelvey, Eric Goldsmith

**Affiliations:** 1 Surgery, Nova Southeastern University Dr. Kiran C. Patel College of Osteopathic Medicine, Fort Lauderdale, USA; 2 Osteopathic Medicine, Nova Southeastern University Dr. Kiran C. Patel College of Osteopathic Medicine, Fort Lauderdale, USA; 3 Medicine, Nova Southeastern University Dr. Kiran C. Patel College of Osteopathic Medicine, Fort Lauderdale, USA; 4 Osteopathic Medicine, Nova Southeastern University Dr. Kiran C. Patel College of Osteopathic Medicine, Clearwater, USA

**Keywords:** do-it-yourself, skills and simulation training, laparascopic surgery, simulation in healthcare, minimally invasive laparoscopy

## Abstract

In 2008, the American Board of Surgery required residents to pass a laparoscopic fundamentals examination to sit for the boards. As such, minimally invasive surgery became the newest addition as a requisite skill for surgical trainees. To assist in preparing trainees for future surgery, simulation devices have been integrated into training programs to develop proficiency with laparoscopic and arthroscopic techniques. While effective, one of the biggest obstacles to accessing these devices is the thousands of dollars required for the equipment. Many commercial and do-it-yourself (DIY) iterations of low-cost, portable, laparoscopic simulators have been described to address this. While the price point ranges from 300 to 400 dollars, these DIY simulators primarily utilize webcams, iPhones, and tablet cameras in a fixed position. This presents an inherent limitation in the simulator’s accuracy as current laparoscopy surgery utilizes camera motion. This study presents a novel DIY simulator that portrays a more realistic view of the operative field using camera motion and positioning, costing approximately 200 dollars.

This proposed simulator uses a Universal Serial Bus (USB) endoscope with interchangeable side mirrors. We inserted an endoscope with built-in light-emitting diode (LED) lights into a seamless stainless tube for the laparoscope and attached it to a computer for configuration. To simulate the abdominal cavity, holes were drilled into a ½ torso hollow mannequin at the standard port locations for laparoscopic cholecystectomy, and rubber grommets were inserted into the drilled holes. Trocars were constructed using cross-linked polyethylene (PEX) tubing and #8 rubber stoppers.

By creating a more affordable and easily constructed model, acquiring laparoscopic skills is more accessible. Simulators are becoming an essential part of medical training. Affordable simulators like ours allow trainees to develop their laparoscopic skill set at their own pace and convenience. More research into this can potentially lead to increased exposure to more accurate simulators and facilitate more accessible training for performing minimally invasive surgery in any surgical specialty.

## Introduction

Minimally invasive surgery is becoming more prevalent than ever in the world of medicine [1]. Despite many areas of medicine performing these types of surgery, the current state of teaching about the techniques and procedure often falls by the wayside [1]. Additionally, most medical schools have shifted toward an anatomy course that is exclusively utilizing prosections in lieu of a full cadaver; this leads to an opportunity lost to learn how to use a scalpel, needle driver, and other surgical tools [2]. Oftentimes, if a student were to be interested in surgical procedures, they would be limited to interest groups and clubs to get a brief tutorial on suturing on pig’s feet and the like [2]. Once on the surgical clerkship rotation of the third year, the medical student must meet a set of expectations, including understanding sterile technique, demonstrating operating room etiquette, and being proficient in simple suturing and knot tying in a matter of weeks [2]. Given the sheer volume of different technology, procedures, and medical knowledge, it is simply not feasible for the medical student to ascertain a sufficient knowledge base of the myriad of suture techniques [2].

While many renditions have attempted to simulate a minimally invasive procedure, there is often a cost burden that is too great for a student to endure [3]. To address this issue, many do-it-yourself (DIY) simulators primarily utilize webcams, smartphones, and tablet cameras in a fixed position to simulate the view of the operative field [3,4]. As an example, one DIY model for minimally invasive laparoscopy utilizes a webcam mounted to a box to simulate the field of view [5]. While the cost burden of these is not nearly that of the commercial simulation training opportunities, the limitation of these current DIY simulators is that the “fixed position” camera perspective is simply not an adequate representation of what occurs in the surgical setting [3]. On the other side of the cost burden, more expensive commercial simulators utilize laparoscopes that accurately simulate the operative field view, which may account for their better face validation when compared to that of DIY models [5]. Therefore, the purpose of this technical report is to present a novel, low-cost DIY laparoscopic simulator that addresses the current limitation of current DIY models by utilizing a Universal Serial Bus (USB) endoscope with interchangeable side mirrors and camera motion plus positioning to provide a more realistic view of the operative field in minimally invasive surgeries.

## Technical report

Designing the scope

**Figure 1:**
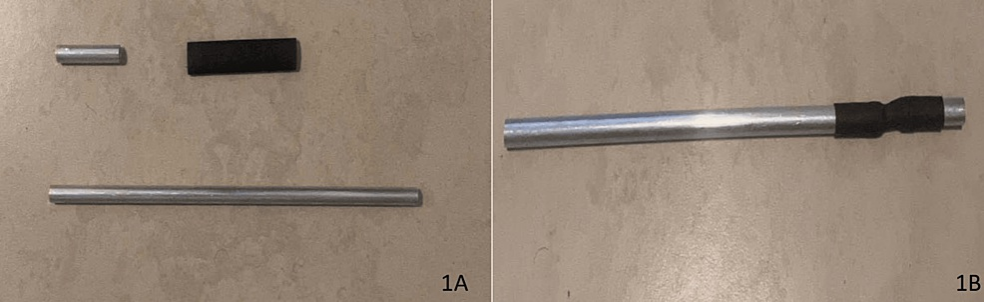
(A) Aluminum tubes and heat shrink. (B) Aluminum tubes joined together by heat shrink.

**Figure 2:**
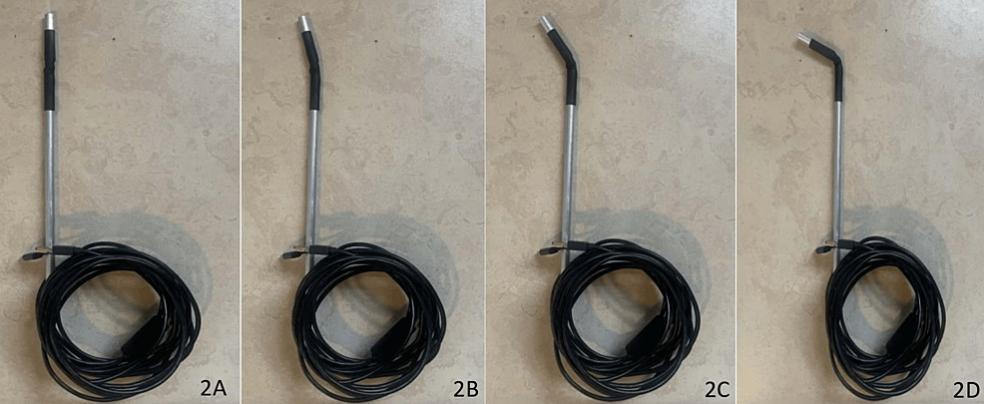
(A-D) Demonstration of various configurations of camera angle due to semirigid design.

Designing the DIY simulator of minimally invasive laparoscopic surgery begins with joining together two aluminum tubes with a length of 12 inches (30.48 cm) and 2 inches (5.08 cm), an internal diameter of 0.234 inches (0.594 cm), and an external diameter of 0.312 inches (0.792 cm). These tubes were joined together by 0.375-inch (0.953 cm) electrical heat shrink (Figure 1A, 1B). Combining them with heat shrink creates a semirigid structure that provides the operator the ability to reconfigure the camera angle to observe the desired operative field (Figure 2).

**Figure 3:**
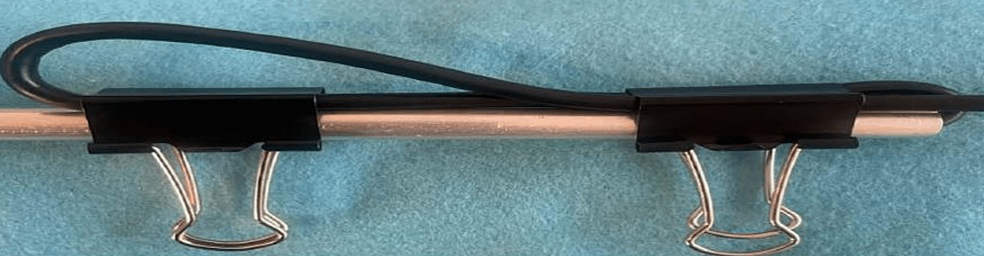
Binder clips locking camera into an upright position.

A 0.0217-inch (0.055 cm) waterproof endoscope with built-in light-emitting diode (LED) lights was inserted into the semirigid structure, and binder clips were used to lock the camera into a fixed position; this ultimately allows for an upright camera image to be maintained (Figure 3). The endoscope utilized a Universal Serial Bus (USB), which was connected and configured to a laptop using a computer-based camera application. This completes the design process of the endoscope and housing.

Designing the operative field

**Figure 4:**
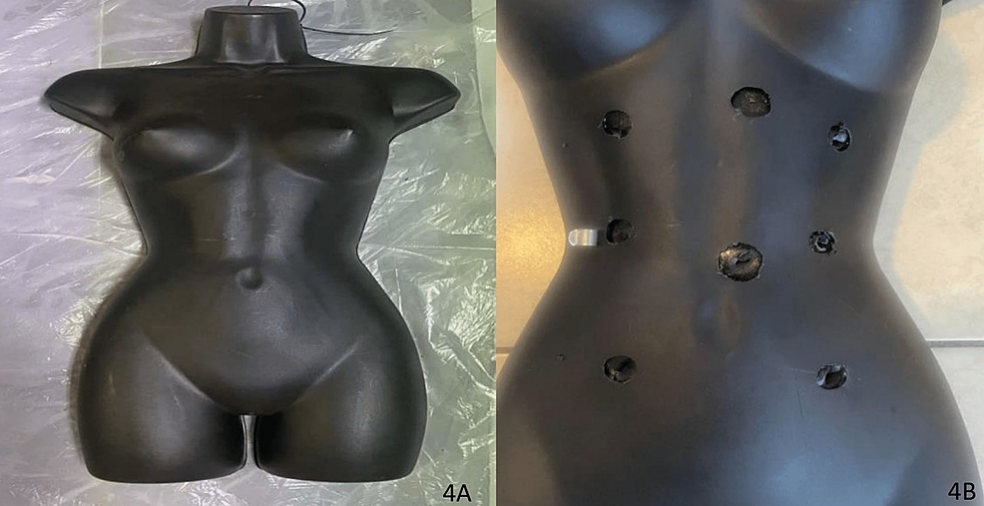
(A) Hollow torso mannequin. (B) Drilled holes in various anatomical locations.

To simulate the abdominal cavity, a total of eight 1.5-inch (3.81 cm) holes were drilled into a hollow torso mannequin (Figure 4A, 4B). A nonslip toolbox liner was glued inside utilizing standard cyanoacrylate adhesive glue. Finally, makeshift port sites were cut out to simulate proper trocar and camera access that one would have while in the operating room.

**Figure 5:**
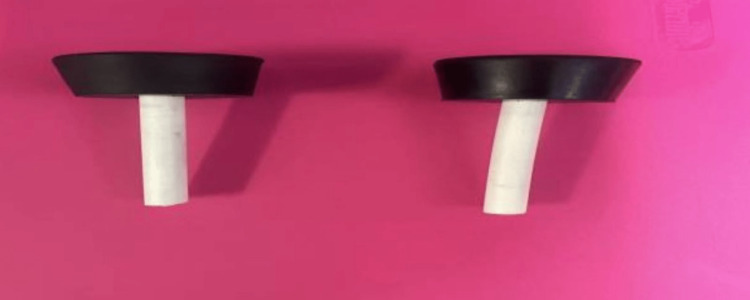
Constructed trocars utilizing #8 rubber stoppers and PEX tubing. PEX: cross-linked polyethylene

Two trocars were constructed using #8 rubber stoppers that have a top diameter of 1.63 inches (4.14 cm), bottom diameter of 1.31 inches (3.33 cm), and height of 1.00 inches (2.54 cm), and cross-linked polyethylene (PEX) tubing with a length of 3 inches (7.62 cm) and a diameter of 0.25 inches (0.635 cm). A 0.375-inch (0.953 cm) hole was drilled into each stopper, and the PEX tubing was inserted without the use of adhesives (Figure 5).

Designing dexterity exercises

**Figure 6:**
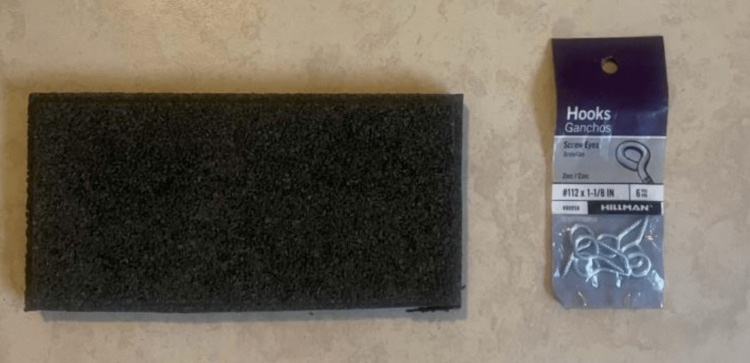
Rubber tile and screw-eye hooks.

**Figure 7:**
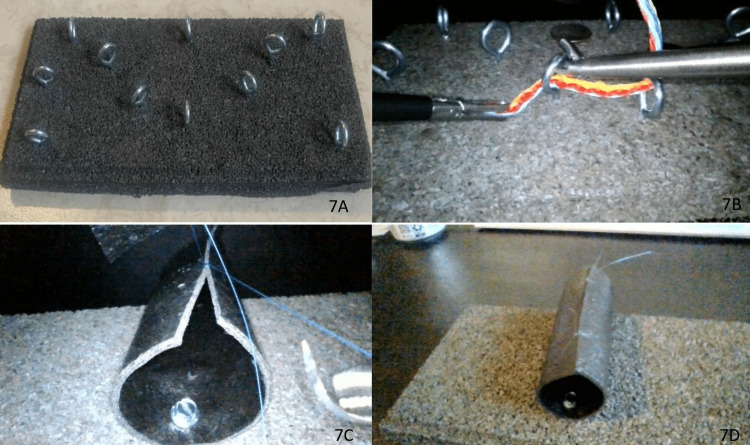
(A) Loop pull-through exercise. (B) Loop pull-through exercise demonstration. (C) Suture exercise. (D) Suturing exercise final product.

As with all laparoscopic simulators, effective practice is the only way the operator can improve dexterity (Figure 6). Here, various exercises were constructed utilizing high-density rubber tile and screw-eye hooks (Figure 7A-7D). Two examples include a loop pull-through exercise series and a suturing exercise.

## Discussion

As technology has advanced, so too has the ability for future surgeons to hone their technical skills. As such, to encourage laparoscopy training, the American Board of Surgery required residents to pass a laparoscopic fundamentals examination to sit for the board since 2008 [3,6]. With the genesis of this new examination, there has been a need for laparoscopic training that can sufficiently simulate it [3]. As with all simulators, there has been a great level of hesitancy and resistance to integrating a laparoscopic one due to the high cost associated with it [3,7]. This program had an overall cost of $11,626, of which $4,967 was earmarked just for equipment and $1,750 for supplies [7]. This burden sparked the advent of creating DIY laparoscopic simulators [3,6].

The basic skeleton of a DIY laparoscopic simulator involves four major components: an encasing to simulate performing surgery on the body habitus, a type of visual monitor, port sites, and a variety of modules on which to practice. While most DIYs will utilize a cardboard box or a plastic container, ours incorporates a mannequin to better simulate the body’s contours [4,5,8-10]. Interestingly, Al-Abed et al. [9] utilize a plastic container with a latex glove covering the opening to simulate the peritoneum. Multiple DIYs have opted to use cameras and mirrors to simulate a two-dimensional work area, but our design incorporates an endoscope connected to a semirigid structure to maximize the vision of the operative field with multiple degrees of freedom [4,5,9,10]. This scope design is semirigid and mobile, allowing the camera angle to be altered according to the operator’s needs. In an effort to enhance the simulator experience, the endoscope used in our design had built-in LED lights; this bypassed the need to include a separate light source in the final design [5,8]. Every laparoscopic simulator had some form of port sites in the encasing used to simulate the body habitus; whether drilled or cut, holes were made to simulate them [4,5,8-10]. Modules were created to assess the functionality of the DIYs assembled [4,9]. Although they may not be the most technically challenging, the modules created were believed to be implementing the most common practices in laparoscopic surgery: loop pull-throughs and suturing. A set of eye hooks were scattered across a high-density rubber tile with the objective being pulling rope through all hooks. They were placed in different orientations to allow for maximum dexterity and practice with altering the position of the graspers. The exercises performed to mimic and practice laparoscopic techniques are entirely customizable and only limited by the operator’s imagination.

A proposed low-cost system was designed to mimic laparoscopic imaging and techniques to provide a way for aspiring surgeons and surgical residents to develop their laparoscopic skills. Table 1 reveals a cost breakdown of the proposed DIY simulator. This designed system has a total cost that is 50% of that of current commercially available scopes with similar optic specifications and operative field perspective [11].

**Table 1:** Cost breakdown to assemble total DIY laparoscopic simulator. DIY: do-it-yourself, LED: light-emitting diode, PEX: cross-linked polyethylene

Consumables to assemble the product	Unit cost
Uxcell 4Pcs 6063 Seamless Aluminum Round Straight Tubing	$3.50
TAKMLY 5.5-mm waterproof endoscope with built-in LED lights	$15.99
Laparoscopy Simulation Training Instruments: Maryland dissector, grasper, scissors, needle holder	$104.00
PEX pipe 1/2” OD length 1’	$0.59
#8 rubber stoppers x 2	$4.98
Craftsman non-slip toolbox liner	$14.99
#1 18” x 18” rubber paver	$5.95
Eye hook	$2.98
Binder clips	$1.00
Optional: hollow mannequin torso	$18.00
User-supplied laptop	Not included
Total cost	$156.99
Total cost without the Laparoscopy Simulation Training Instruments	$52.99

To assess how the proposed simulator compares to other DIY designs, Table 2 includes different proposed DIY designs along with the cost, benefits, and any major limitations. It should be noted that the DIYs did not include the cost of the laparoscopy simulation training equipment (Maryland dissector, grasper, scissors, needle holder) [4,5,8-10]. Removing this cost from our build brings the cost to a cool $52.99. Limitations associated with this DIY system are the image and lighting quality compared to actual laparoscopic equipment and the high price of surgical instruments comprising most of the material cost. Further research is required to confirm the proposed construct’s validity as a competitive teaching product that can be utilized. If validated, additional research is recommended to finetune and create an even more realistic and low-cost DIY laparoscopic training system that aspiring surgeons and surgical residents can utilize to develop their laparoscopic skills.

**Table 2:** Other proposed DIY designs along with the cost, benefits, and any major limitations. DIY: do-it-yourself

Design	Cost (US dollars)	Benefits	Limitations
Sellers et al. [4]	$58.98	Cost-effective, created modules associated with it, no separate light source	Plastic box to simulate body habitus, more challenging to assemble
Khine et al. [5]	$74.07	Cost-effective, skin-like access ports	Separate external light source, plastic container to simulate body habitus, no created modules associated with it
Chung et al. [8]	Not disclosed	Created modules associated with it, endoscopic camera utilized	Separate external light source, fiberglass container to simulate body habitus
Al-Abed et al. [9]	$49.83	Cost-effective, easy to assemble, simulates penetrating peritoneum, no external light source	Plastic box to simulate body habitus

## Conclusions

Laparoscopic surgery has become one of the mainstay modalities to perform surgery. In all surgical specialties, mastery of laparoscopy is paramount. In an effort to assist the next generation of surgeons in their practice, we set out to create a realistic low-cost do-it-yourself laparoscopic simulator with just a few materials that one could find at a local hardware store. Ultimately, it is our hope that this can one day be used to help residents hone their technical skills and instill in medical students a deeper appreciation for laparoscopic surgery.
